# Stable Isotope Tracing Reveals an Altered Fate of Glucose in *N*-Acetyltransferase 1 Knockout Breast Cancer Cells

**DOI:** 10.3390/genes14040843

**Published:** 2023-03-31

**Authors:** James T. F. Wise, Xinmin Yin, Xipeng Ma, Xiang Zhang, David W. Hein

**Affiliations:** 1 Department of Pharmacology and Toxicology, School of Medicine, University of Louisville, Louisville, KY 40202, USA; jamest.wise@louisville.edu; 2 Department of Chemistry, University of Louisville, Louisville, KY 40292, USA; xinmin.yin@louisville.edu (X.Y.); xipeng.ma@louisville.edu (X.M.); xiang.zhang@louisville.edu (X.Z.); 3 Center for Regulatory and Environmental Analytical Metabolomics, University of Louisville, Louisville, KY 40292, USA

**Keywords:** *N*-acetyltransferase 1, breast cancer, stable isotope tracing, metabolism, mitochondrial metabolism, breast cancer cells

## Abstract

Breast cancer is one of the leading causes of cancer death. Recent studies found that arylamine *N*-acetyltransferase 1 (NAT1) is frequently upregulated in breast cancer, further suggesting NAT1 could be a potential therapeutic target for breast cancer. Previous publications have established that *NAT1* knockout (KO) in breast cancer cell lines leads to growth reduction both in vitro and in vivo and metabolic changes. These reports suggest that NAT1 contributes to the energy metabolism of breast cancer cells. Proteomic analysis and non-targeted metabolomics suggested that *NAT1* KO may change the fate of glucose as it relates to the TCA/KREB cycle of the mitochondria of breast cancer cells. In this current study, we used [U-^13^C]-glucose stable isotope resolved metabolomics to determine the effect of *NAT1* KO on the metabolic profile of MDA-MB-231 breast cancer cells. We incubated breast cancer cells (MDA-MB-231 cells) and *NAT1* Crispr KO cells (KO#2 and KO#5) with [U-^13^C]-glucose for 24 h. Tracer incubation polar metabolites from the cells were extracted and analyzed by 2DLC-MS, and metabolite differences were compared between the parental and *NAT1* KO cells. Differences consistent between the two KO cells were considered changes due to the loss of NAT1. The data revealed decreases in the ^13^C enrichment of TCA/Krebs cycle intermediates in *NAT1* KO cells compared to the MDA-MB-231 cells. Specifically, ^13^C-labeled citrate, isocitrate, a-ketoglutarate, fumarate, and malate were all decreased in *NAT1* KO cells. We also detected increased ^13^C-labeled *L*-lactate levels in the *NAT1* KO cells and decreased ^13^C enrichment in some nucleotides. Pathway analysis showed that arginine biosynthesis, alanine, aspartate and glutamate metabolism, and the TCA cycle were most affected. These data provide additional evidence supporting the impacts of *NAT1* knockout on cellular energy metabolism. The data suggest that NAT1 expression is important for the proper functioning of mitochondria and the flux of glucose through the TCA/Krebs cycle in breast cancer cells. The metabolism changes in the fate of glucose in *NAT1* KO breast cancer cells offer more insight into the role of NAT1 in energy metabolism and the growth of breast cancer cells. These data provide additional evidence that NAT1 may be a useful therapeutic target for breast cancer.

## 1. Introduction

Arylamine *N*-acetyltransferase 1 (NAT1) is a phase II metabolic enzyme that uses acetyl coenzyme A to acetylate drugs and xenobiotics [1,2,3]. NAT1 is found in almost all human tissues [1,2,3]. In addition to the role of NAT1 in xenobiotic metabolism, recent publications have implicated NAT1 in other biological roles. NAT1 upregulation occurs in both estrogen receptor-positive and triple-negative breast cancers [4,5,6,7]. This upregulation of NAT1 in breast cancers led to investigations into understanding the role of NAT1 in cancer cell energetics, cell growth, and cell morphology [8,9,10,11,12,13,14,15,16]. However, the exact molecular and cellular importance of NAT1 expression in breast cancer remains elusive.

Studies have utilized both small molecule-mediated inhibition and CRISPR/Cas9 *NAT1* KO to investigate the role of NAT1 in breast cancer cell lines. Although there have been some discrepancies reported between studies, there is consensus that NAT1 inhibition or *NAT1* KO in cultured breast cancer cells results in cell growth retardation and reduced migration/invasion [14,15,16,17,18,19,20]. Further, the inhibition or loss of NAT1 results in a loss of the ability of breast cancer cells to grow in an anchorage-independent manner (growth in soft agar). *NAT1* KO in MDA-MB-231 breast cancer cells reduced anchorage-independent colony formation and primary and secondary tumors in immunocompromised mice [14,15,17,19,20].

Examinations into the role of NAT1 in breast cancer cellular energetics have also been investigated. Proteomic and untargeted metabolomics data indicated that NAT1 negatively impacted the mitochondria, but inconsistent results were reported between mitochondrial respiration endpoints as measured by the Seahorse Analyzer in MDA-MB-231 cells [9,16]. Both studies reported that *NAT1* knockout in breast cancer cells increased glycolytic activity. Given the results of these studies, the data suggest changes in the fate of glucose resulting from *NAT1* KO. To build on our previous results and the results of others, the objective of our study was to use stable isotope tracing to track the fate of glucose in *NAT1* KO breast cancer cells and to provide more insight into the impacts of *NAT1* KO on breast cancer cell mitochondria and the role of NAT1 in the cellular energetics of breast cancer cells.

## 2. Materials and Methods

### 2.1. Reagents

Acetic acid, DMSO, methanol, PBS, para-aminobenzoic acid (PABA), 4-acetamidobenzoic acid (*N*-acetyl-PABA), D-glucose, and sodium perchlorate were purchased from Millipore-Sigma (St. Louis, MO, USA). Trypsin/EDTA, PBS, DMEM, *L*-glutamine, Pen-Strep, and culture ware were purchased from Thermo Fisher Scientific (Carlsbad, CA, USA). [U-^13^C]-glucose was purchased from Cambridge Isotope Laboratories, Inc. (Tewksbury, MA, USA).

### 2.2. Cell Culture

*NAT1* KO MDA-MB-231 and MDA-MB-231 (scramble, i.e., nonspecific scrambled shRNA inserted in an FRT site, referred to as MDA-MB-231) cell lines were used as previously described [15]. *NAT1* “KO2” and “KO5” cells represent the two different KO cell lines generated using two unique guide RNAs and CRISPR/Cas9. MDA-MB-231 and *NAT1* KO MDA-MB-231 cells were cultured in DMEM media (high glucose: 4.5 g/L), fetal bovine serum (10%), *L*-glutamine (4 mM), sodium pyruvate (1 mM), and pen/strep (1%). Cells were maintained at 37 °C with 5% CO_2_.

### 2.3. Measurement of N-Acetylation of PABA

Cells were incubated with PABA (50 µM) for 48 h in culture media. After 48 h of incubation, the media were collected in centrifuge tubes with 1/10 volume of 1 M acetic acid and then centrifuged at 15,000 *g* for 10 min. Cells were counted using Beckman Coulter Z1 DUAL (Beckman Coulter, Inc., Brea, CA, USA), and the data used were normalized for the number of cells. *N*-acetylated PABA in the culture media was separated and quantitated using HPLC (Agilent Technologies 1260 Infinity) for PABA as described previously [21]. The HPLC limit of detection was 0.005 nmol PABA [15].

### 2.4. [U-^13^C]-Glucose Tracer Studies

Cells were seeded at 8 × 10^5^ cells in 10 cm dishes and allowed a 24 h rest. Cells were then labeled for 24 h with DMEM (Gibco, Cat. No. A14430-01) supplemented with 1 g/L [U-^13^C]-glucose (Cambridge Isotopes, Cat. No. CLM-1396-1) or [U-^12^C]-glucose (Sigma, Cat. No. G7021-100G) and 10% dialyzed fetal bovine serum (HyClone, Cat. No. AE29416440), 4 mM *L*-glutamine (Corning, Cat. No. 25-005-Cl), 1 mM sodium pyruvate (HyClone, Cat. No. SH30239.01), and 1% penicillin-streptomycin (HyClone, Cat. No. SV0010). There were four plates per cell line per experiment (two plates with [U-^13^C]-glucose and two with [U-^13^C]-glucose). One plate for each glucose type was used for metabolites, and one plate for extractions. Metabolites were extracted using a modification of previously published methods [22]. In brief, cells were washed three times with ice-cold PBS. Next, cells were quenched with 1.5 mL of cold (−20 °C) acetonitrile on each plate. Polar metabolites were extracted in acetonitrile: water (3 mL:750 µL). After centrifugation at 14,000 rpm for 20 min at 4 °C, the supernatant was transferred into a new tube and lyophilized overnight. The dried sample was reconstituted in 60 µL 50% CH_3_CN for 2DLC-MS analysis.

Detailed 2DLC-MS setting information is provided as Appendix A. Briefly, all samples were analyzed by parallel two-dimensional liquid chromatography-mass spectrometry (2DLC-MS) composed of a Thermo Q Exactive HF Hybrid Quadrupole-Orbitrap Mass Spectrometer coupled with a Thermo DIONEX Ultimate 3000 HPLC system (Thermo Fisher Scientific, Waltham, MA, USA). The samples were separated on a reversed phase chromatography (RPC) column and a hydrophilic interaction chromatography (HILIC) column, respectively. To obtain full MS data, each sample was analyzed by the 2DLC-MS in both positive (+) and negative (−) mode. For metabolite identification, the group-based pooled samples were analyzed by 2DLC-MS/MS in positive mode and negative mode to acquire MS/MS spectra. Isotopologue identification, spectrum deconvolution, and cross-sample peak list alignment were reported in our previous publications [23,24,25]. All metabolites detected are provided in the Supplementary Materials Tables S1–S3, and the statistical results are presented in Section 3.

### 2.5. Statistical Analysis

Statistical analyses were performed using SPSS software (version 25, IBM Corporation, Armonk, NY, USA). Distributional assumptions of continuous outcomes were checked, and, if needed, a data transformation (e.g., a log-transformation) was applied to meet the normality assumption. Univariate analysis of isotopologue abundance among groups was conducted using a one-way ANOVA with a Tukey post-hoc test, and the Benjamini & Hochberg method was used for multiple testing correction [26]. Statistical significances between any groups are as follows: * *q*  <  0.05, ** *q*  <  0.01, and *** *q*  <  0.001. The error bars in each histogram plot are the standard error of the mean (SEM).

## 3. Results

### 3.1. NAT1 Knockout Does Not Alter the Glycolytic Intermediates in MDA-MB-231 Cells

**Figure 1:**
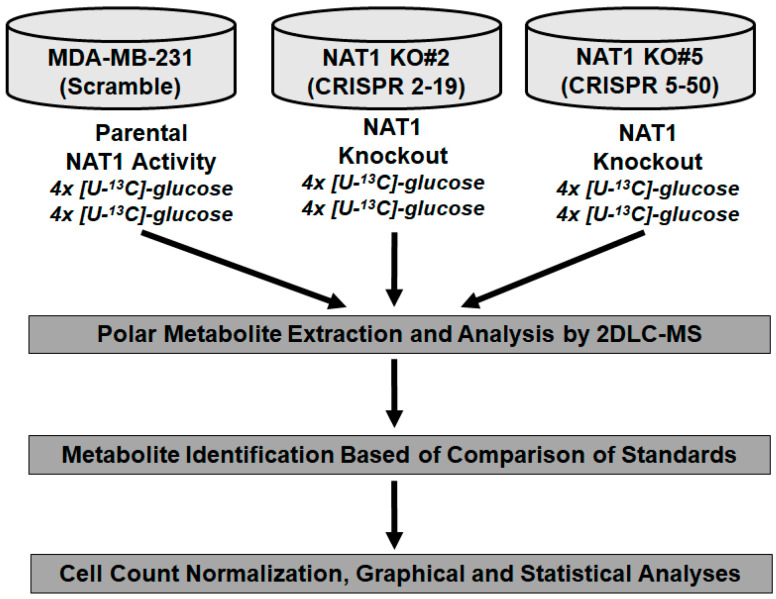
**Schematic of Experimental Approach.** Four biological replicates from each cell line were collected for both [U-^13^C]- and [U-^12^C]-glucose. Samples were analyzed by 2DLC-MS/MS. Following metabolite identification, data was normalized to cell counts and analyzed for consistent statistical differences between *NAT1* KO and MDA-MB-231 (scramble) cells.

**Figure 2:**
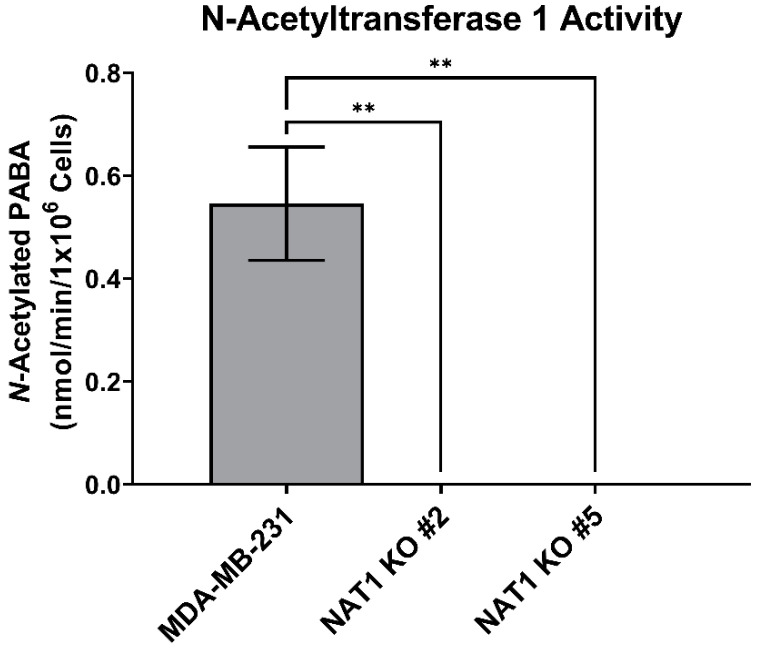
***N*-Acetyltransferase 1 Knockout Effect on *N*-Acetylation of PABA by Breast Cancer Cells**. *N*-acetylated PABA levels in MDA-MB-231 cells and *NAT1* KO #2 and #5 after 48 h incubation with 50 µM PABA. Data represent the average of at least three experiments ± SEM; ** *p* <  0.01 compared to MDA-MB-231.

**Figure 3:**
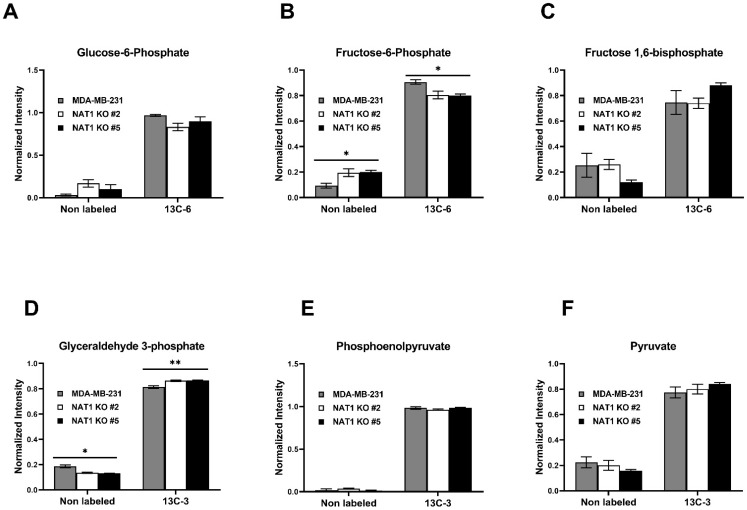
***N*-Acetyltransferase 1 Knockout Effect on the Glycolytic Intermediates in MDA-MB-231 Cells.** Carbon-13 enrichment of glycolytic intermediates was measured in MDA-MB-231 cells and *NAT1* KO #2 and #5 after 24 h incubation with [^13^C]-glucose. Data are presented for both unlabeled [^12^C] and detected [^13^C]-labeled metabolites (**A**) Glucose-6-Phosphate (^13^C-6) (**B**) Fructose-6-Phosphate (^13^C-6) (**C**) Fructose 1,6-Bisphosphate (^13^C-6) (**D**) Glyceraldehyde 3-Phosphate (^13^C-3) (**E**) Phosphoenolpyruvate (^13^C-3) (**F**) Pyruvate (^13^C-3). Data represent average of at least 3 experiments ± SEM, * *q* <  0.05 and ** *q*  <  0.01 compared to MDA-MB-231.

**Figure 4:**
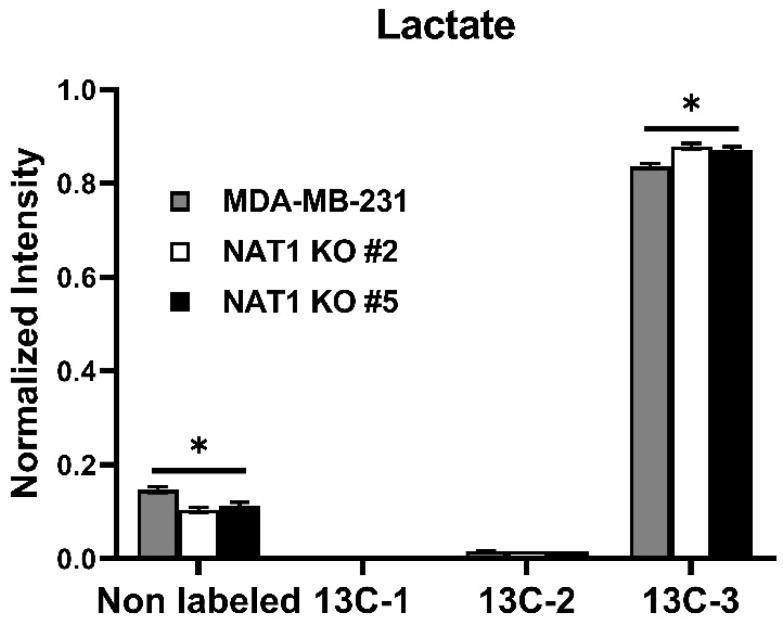
***N*-Acetyltransferase 1 Knockout Effect on the *L*-Lactate Levels of MDA-MB-231 Cells.** Carbon-13 enrichment of *L*-Lactate was measured in MDA-MB-231 cells and *NAT1* KO #2 and #5 after 24 h incubation with [^13^C]-glucose. Data presented for both unlabeled [^12^C] and detected [13C]-labeled metabolites (^13^C-1, ^13^C-2, and ^13^C-3). Data represent the average of at least three experiments ± SEM, *q <  0.05 compared to MDA-MB-231.

To investigate the metabolic changes to glucose utilization following *NAT1* KO in breast cancer cells, we used [U-^13^C]-glucose tracer studies. The experimental schematic is presented in Figure 1. The cell lines used were MDA-MB-231 cells and two Crispr/Cas9 *NAT1* KO clones (#2 and #5) that were previously described [15]. The *NAT1* KO cells used showed a complete loss of the *N*-acetylation of the prototypical NAT1 substrate, PABA (Figure 2). We did not observe a consistent major trend in the [^13^C]-glycolytic intermediates in the *NAT1* KO cells compared to the MDA-MB-231 cells (Figure 3). Specifically, glucose-6-phosphate, fructose 1,6-bisphosphate, phosphoenolpyruvate, and pyruvate levels for both ^12^C and ^13^C isotopologues were unchanged between *NAT1* KOs and MDA-MB-231. The fructose-6-phosphate levels (^13^C-6) were decreased in both *NAT1* KO cells compared to MDA-MB-231 cells (*q* < 0.05), whereas glyceraldehyde-3-phosphate (^13^C-3) was increased in both *NAT1* KO#2 and KO#5 compared to MDA-MB-231 (*q* < 0.01). Other intermediates in the *NAT1* KO cells were unchanged compared to MDA-MB-231 cells. Thus, we conclude the fate of glucose to pyruvate in the MDA-MB-231 cells was relatively unaffected by *NAT1* KO. However, the level of [^13^C]-lactate (^13^C-3) was increased in the *NAT1* KO cell lines (*q* < 0.05) (Figure 4).

### 3.2. NAT1 Knockout Reduces the TCA/Krebs Cycle Intermediates of MDA-MB-231 Cells

**Figure 5:**
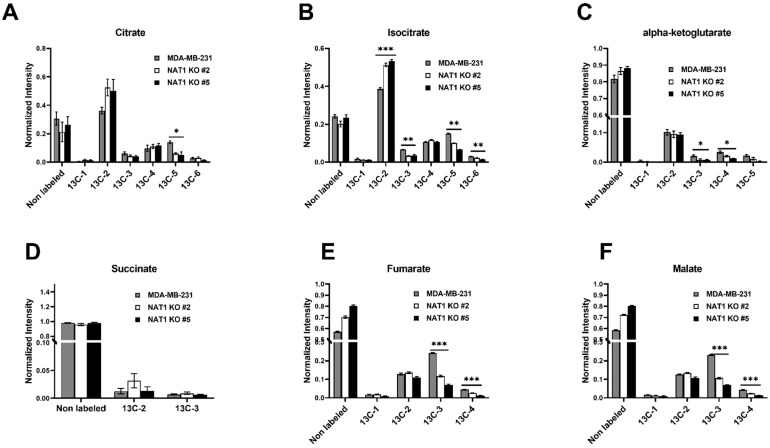
***N*-Acetyltransferase 1 Knockout Effect on the TCA/Krebs Cycle Intermediates in MDA-MB-231 Cells.** Carbon-13 enrichment of TCA/Krebs cycle intermediates were measured in MDA-MB-231 cells and *NAT1* KO #2 and #5 after 24 h incubation with [^13^C]-glucose. Data are presented for both unlabeled [^12^C] and detected [13C]-labeled metabolites. (**A**) Citrate (^13^C-1,-2,-3,-4,-5, and -6) (**B**) Isocitrate (^13^C-1,-2,-3,-4,-5, and -6) (**C**) α-Ketoglutarate (^13^C-1,-2,-3,-4, and -5) (**D**) Succinate (^13^C-2 and ^13^C-2) (**E**) Fumarate (^13^C-1,-2,-3, and -4) (**F**) Malate (^13^C-1,-2,-3, and -4). Data represent the average of at least 3 experiments ± SEM, * *q*  <  0.05, ** *q* <  0.01, and *** *q*  <  0.001 compared to MDA-MB-231.

We then examined whether glucose metabolism within other metabolism pathways, such as the TCA/Krebs cycle, was altered by *NAT1* KO. We observed decreases in overall enrichment of [^13^C]-citrate, -isocitrate, -α-ketoglutarate, -succinate, -fumarate, and -malate levels in the *NAT1* KO cells compared to the MDA-MB-231 cells (Figure 5). Interestingly for isocitrate, ^13^C-2 was increased in *NAT1* KO, but the rest of the ^13^C-labeled carbons [^13^C-3, -5, -6] were decreased (Figure 5B). Thus, the overall trend is a decrease in ^13^C enrichment into isocitrate. Further, in both *NAT1* KO cells, we observed decreases in [^13^C-5]-citrate, [^13^C-3, -5, -6]-isocitrate, [^13^C-3, -4]-α-ketoglutarate, [^13^C-3, -4]-fumarate, and [^13^C-3, -4]-malate that were all statistically significant (Figure 5). These data indicate less glucose flux through the TCA/Krebs cycle with *NAT1* KO.

### 3.3. NAT1 Knockout Alters the Amino Acid Production of MDA-MB-231 Cells

**Figure 6:**
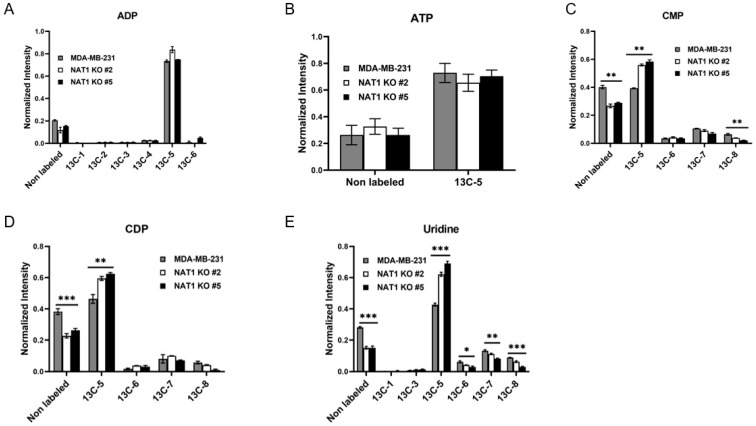
***N*-Acetyltransferase 1 Knockout Effect on Nucleotides in MDA-MB-231 Cells.** Carbon-13 enrichment of nucleotides were measured in MDA-MB-231 cells and NAT1 KO #2 and #5 after 24 h incubation with [^13^C]-glucose. Data are presented for both unlabeled [^12^C] and detected [^13^C]-labeled metabolites. (**A**) ADP (^13^C-1,-2,-3,-4,-5,- and 6) (**B**) ATP (^13^C-5) (**C**) CMP (^13^C-5,-6,-7, and -8) (**D**) CDP (^13^C-5,-6,-7, and -8) (**E**) Uridine (^13^C-1,-3,-5,-6,-7, and -8). Data represent the average of at least three experiments ± SEM, * *q*  <  0.05, ** *q*  <  0.01, and *** *q*  <  0.001 compared to MDA-MB-231.

Next, we investigated whether *NAT1* KO altered the amino acid production of MDA-MB-231 cells. We found that [13C]-ATP and -ADP levels were unchanged in *NAT1* KO cells compared to MDA-MB-231 (Figure 6A,B). [^13^C] -CMP (^13^C-5 and ^13^C-8), -CDP (^13^C-5), and -uridine (^13^C-6, -7, and -8) levels were all decreased in NAT1 KO cells compared to the MDA-MB-231 cells (Figure 6). Interestingly, for uridine, while ^13^C-5 was increased, ^13^C-6, -7, and -8 enrichment was decreased. Thus, the overall trend was decreased ^13^C enrichment in uridine. These data demonstrate a decreased flux of [^13^C]-glucose into some amino acids in *NAT1* KO cells and an increased flux of [^13^C]-glucose into other amino acids.

### 3.4. NAT1 Knockout Alters the S-Adenosyl Methionine (SAM) Cycle of MDA-MB-231 Cells

**Figure 7:**
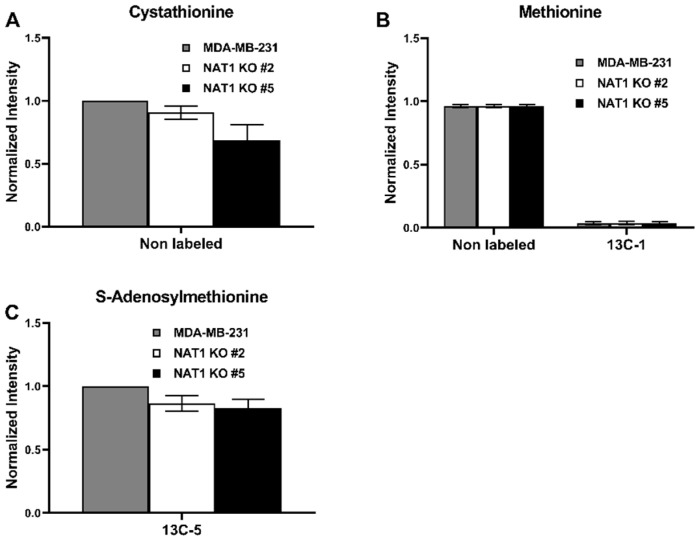
***N*-Acetyltransferase 1 Knockout Does Not Impact the S-Adenosyl Methionine Cycle Intermediates in MDA-MB-231 Cells.** Carbon-13 enrichment of S-Adenosyl Methionine Cycle intermediates were measured in MDA-MB-231 cells and *NAT1* KO #2 and #5 after 24 h incubation with [^13^C]-glucose. Data are presented for both detected unlabeled [^12^C] and [^13^C]-labeled metabolites. (**A**) Cystathionine (**B**) Methionine (^13^C-1) (**C**) S-Adenosylmethionine (^13^C-5). Data represent the average of at least three experiments ± SEM, no isotopologue has significant abundance changes compared to MDA-MB-231.

Next, we explored the glucose metabolism within the SAM cycle in *NAT1* KO cells. We found that methionine levels were unchanged between all three cell lines (Figure 7B). While [^13^C]-cystathionine in *NAT1* KO cells compared to MDA-MB-231 cells, and [^13^C]-S-adenosylmethionine levels were decreased in *NAT1* KO compared to MDA-MB-231 cells, but this result was not statistically significant (Figure 7A,C).

### 3.5. NAT1 Knockout Alters the Amino Acid Production of MDA-MB-231 Cells

**Figure 8:**
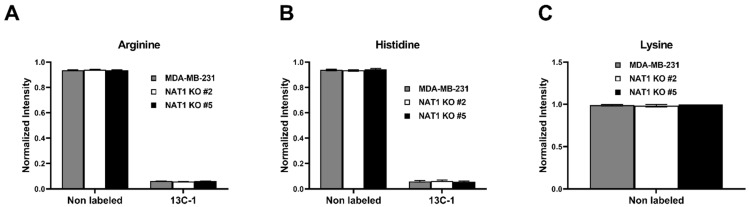

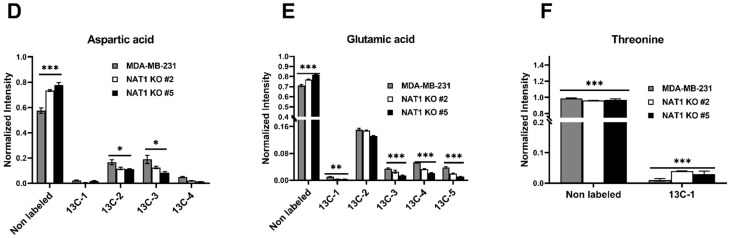
***N*-Acetyltransferase 1 Knockout Effect on Amino Acid Levels in MDA-MB-231 Cells.** Carbon-13 enrichment of charged amino acids were measured in MDA-MB-231 cells and *NAT1* KO #2 and #5 after 24 h incubation with [^13^C]-glucose. Data are presented for both detected unlabeled [^12^C] and [^13^C]-labeled metabolites. (**A**) Arginine (^13^C-1) (**B**) Histidine (^13^C-1) (**C**) Lysine (**D**) Aspartic Acid (^13^C-1,-2,-3, and -4) (**E**) Glutamic Acid (^13^C-1,-2,-3,-4, and -5) (**F**) Threonine (^13^C-1). Data represent the average of at least three experiments ± SEM, * *q*  <  0.05, ** *q*  <  0.01, and *** *q*  <  0.001 compared to MDA-MB-231.

**Figure 9:**
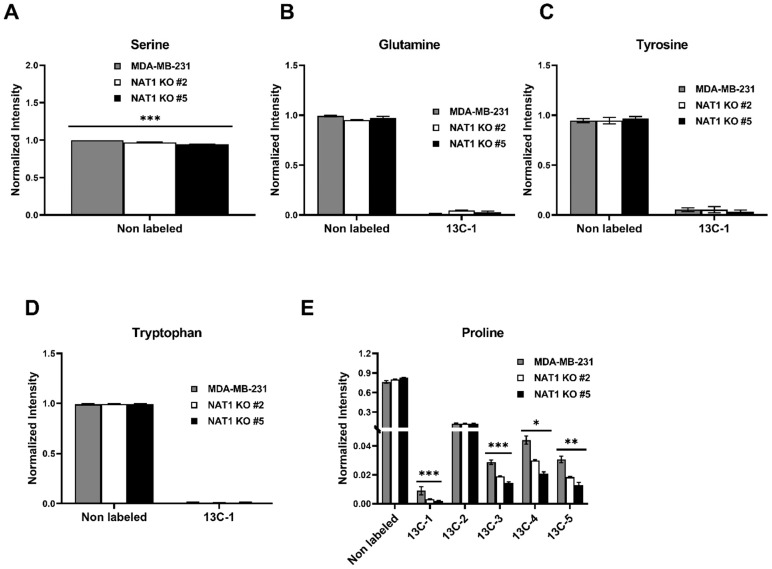
***N*-Acetyltransferase 1 Knockout Effect on Amino Acid Levels in MDA-MB-231 Cells.** Carbon-13 enrichment of charged amino acids were measured in MDA-MB-231 cells and *NAT1* KO #2 and #5 after 24 h incubation with [^13^C]-glucose. Data are presented for both detected unlabeled [^12^C] and [^13^C]-labeled metabolites. (**A**) Serine (**B**) Glutamine (^13^C-1) (**C**) Tyrosine (^13^C-1) (**D**) Tryptophan (^13^C-1) (**E**) Proline (^13^C-1,-2,-3,-4,-5). Data represent the average of at least three experiments ± SEM, * *q* <  0.05, ** *q*  <  0.01, and *** *q* <  0.001 compared to MDA-MB-231.

Lastly, we investigated the glucose flux through amino acid production in *NAT1* KO cells compared to MDA-MB-231 cells (Figure 8 and Figure 9). We observed no changes to arginine, histidine, or lysine levels in *NAT1* KO compared to MDA-MB-231 (Figure 8A–C). Statistical decreases were observed in [^13^C]-aspartic acid (^13^C-2, -3), and -glutamic acid (^13^C-1, -3, -4, -5) in *NAT1* KO cells compared to MDA-MB-231 cells (Figure 8D,E). Interestingly, there was a small increase in [^13^C]-threonine (^13^C-1). The levels of [^12^C]-serine were decreased slightly in the *NAT1* KO cells compared to MDA-MB-231 cells (Figure 9A). Glutamine (^13^C-1) showed no significant consistent changes between the two *NAT1* KO cells and MDA-MB-231 (Figure 9B). [^13^C]-tyrosine and -tryptophan levels were unaffected in *NAT1* KO cells compared to MDA-MB-231 cells (Figure 9C,D). In *NAT1* KO cells, [^13^C]-proline (^13^C-1, -3, -4, and -5) levels were decreased in *NAT1* KO cells compared to MDA-MB-231 cells (Figure 9E).

## 4. Discussion

Our results further support the literature that *NAT1* KO in breast cancer cells alters their cellular metabolism. Further, the present study demonstrated that *NAT1* KO in breast cancer cells (MDA-MB-231) resulted in an altered fate in the metabolism of glucose. The metabolism pathways impacted by the loss of NAT1 in their glucose utilization were lactate production, nucleotides, the TCA/Krebs cycle, alanine, aspartate, and glutamate metabolism. These data provide further evidence of other cellular energetic pathways that are impacted by the loss of NAT1 in breast cancer cells.

A proteomics study suggested that *NAT1* KO cells may reduce the de novo synthesis of pyrimidines [18]. Our results presented in Figure 5 support the hypothesis that de novo synthesis of the pyrimidines may be reduced by *NAT1* KO. Specifically, we observed that uridine synthesis is likely reduced based on ^13^C enrichment of uridine, but the results of ^13^C enrichment into cytosine metabolites were less clear, and cytosine metabolism changes warrant additional investigation. These results and previous published results suggest that breast cancer treatments may be more effective with simultaneous NAT1 inhibition [18]. However, this hypothesis requires additional studies involving more mechanistic in vitro and in vivo models using *NAT1* knockout and cytotoxic nucleosides concomitantly for breast cancer treatments.

Two groups reported an increase in glycolytic activity in *NAT1* KO MDA-MB-231 cells as measured by the Seahorse Analyzer, suggesting the fate of glucose in these NAT1 KO leaned towards more anaerobic glycolysis and the ultimate production of *L*-lactate [9,16]. Our results in Figure 3 highlight that the fate of glucose is shifted slightly towards more *L*-lactate in *NAT1* KO cells, which would result in increased glycolytic activity and a pH shift as previously measured by the Seahorse Analyzer.

As outlined previously, there were changes to the amino acid composition of NAT1 breast cancer cells following non-targeted metabolomics [10]. Our results demonstrate that the levels of aspartic acid and glutamic acid coming from glucose metabolism are affected by *NAT1* KO (Figure 7 and Figure 8). Given the importance of these amino acids to cellular growth and function, it is possible that the reduction observed in these amino acids may have a role in the decreased growth properties of *NAT1* KO cells [18]. However, the overall implications of amino acid metabolism in these *NAT1* KO cells warrant additional investigations. Interestingly, we also observed changes in the ^13^C-enrichment of glucose into uridine diphosphate *N*-acetylglucosamine (UDP-GlcNAc) (Supplementary Table S2), a key metabolite in the hexosamine biosynthesis pathway. These changes to UDP-GlcNAc metabolism also may impact the growth of *NAT1* KO cells compared to MDA-MB-231 cells, but more studies are required.

Multiple independent research groups have reported that inhibition of NAT1 in various breast cancer cell lines resulted in a changed mitochondrial respiration response. Carlisle et al. first reported that *NAT1* KO in MDA-MB-231 cells resulted in an increase in mitochondrial respiration [9]. Conversely, Wang et al. reported that *NAT1* KO in MDA-MB-231 cells resulted in a decrease in mitochondrial respiration [16]. These conflicting impacts of *NAT1* KO on mitochondrial respiration may be due to differences in the Crispr/Cas9 KO methods or the Seahorse Analyzer conditions used. Rather than repeat the same endpoints, we looked to further understand changes related to mitochondrial-related metabolism in breast cancer cells. Further, additional investigations by Carlisle et al. and Hong et al. indicated that there should be a decrease in mitochondrial functions due to changes in the fate of glucose, TCA/Krebs cycle intermediates, and ATP synthase subunits [8,11,18]. Therefore, the goal of this study was to track the fate of glucose and provide clarity to the discrepancy of the mitochondrial respiration endpoints. Based on the data presented here and the current literature on *NAT1* KO in breast cancer cells, we believe that mitochondrial function is reduced by *NAT1* KO and further that the fate of glucose is altered, resulting in less glucose flux through the TCA/Krebs cycle.

**Figure 10:**
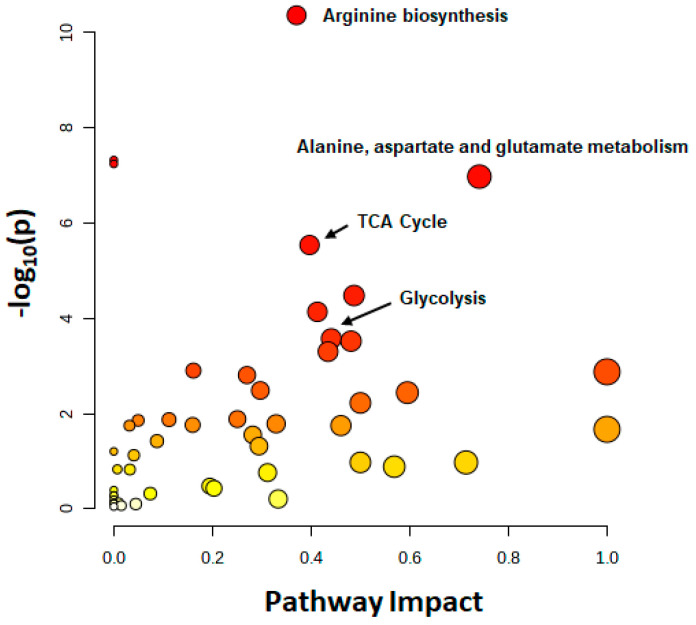
**Metabolites Affected in Their ^13^C-Enrichment from Glucose by *NAT1* KO in Breast Cancer Cells**. Metabolic pathways affected by *NAT1* KO were detected by the culture of MDA-MB-231 breast cancer cells with [U-^13^C]-glucose for 24 h. Results of quantitative pathway enrichment analysis of metabolites detected by 2DLC-MS. The arginine biosynthesis, alanine, aspartate and glutamate metabolism, and the TCA cycle were the most affected pathways.

**Figure 11:**
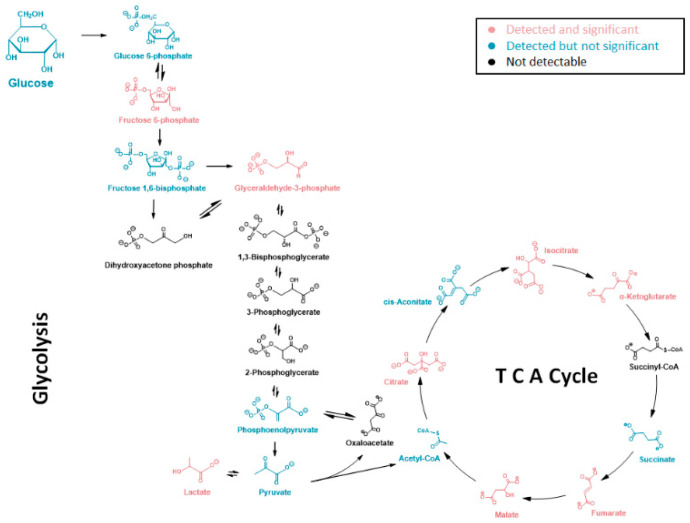
**Glycolytic and TCA/Krebs Cycle Metabolites Affected in Their ^13^C-Enrichment from Glucose by *NAT1* KO in Breast Cancer Cells.** Metabolites were detected in the glycolysis and TCA cycle pathways. Metabolites in black were not detected in this study, while the isotopologues of metabolites in pink showed significant abundance changes from *NAT1* KO. Isotopologues of the metabolites in blue did not show significant abundance changes.

**Figure 12:**
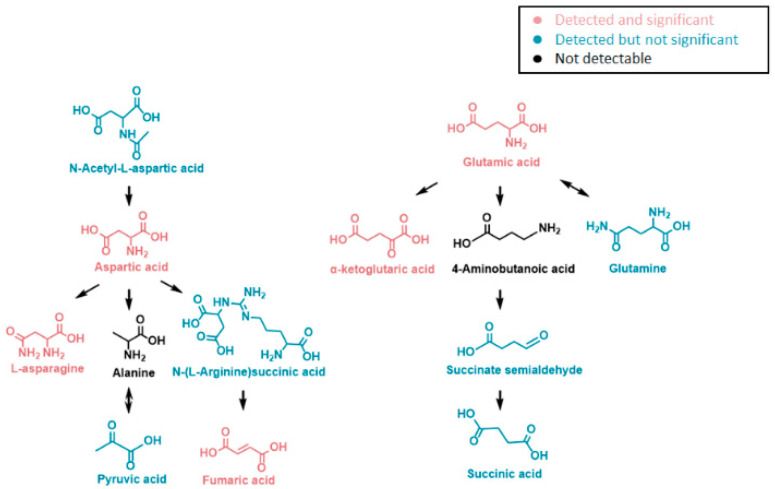
**Alanine, Aspartate, and Glutamate Metabolites Affected in Their ^13^C-Enrichment from Glucose by *NAT1* KO in Breast Cancer Cells.** Metabolites were detected in the alanine, aspartate, and glutamate biosynthesis pathways. Metabolites in black were not detected in this study, while the isotopologues of metabolites in pink showed significant abundance changes from *NAT1* KO. Isotopologues of the metabolites in blue did not show significant abundance changes.

**Figure 13:**
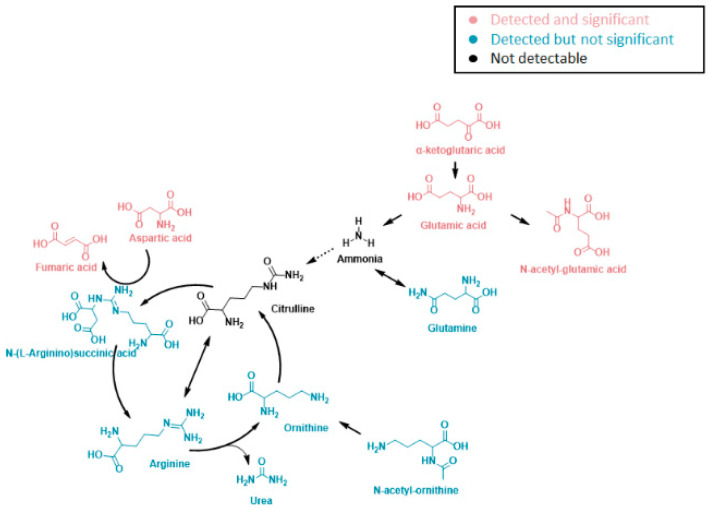
**Arginine Metabolites Affected in Their ^13^C-Enrichment from Glucose by NAT1 KO in Breast Cancer Cells.** Metabolites detected in the arginine biosynthesis pathway. Metabolites in black were not detected in this study, while the isotopologues of metabolites in pink showed significant abundance changes from *NAT1* KO. Isotopologues of the metabolites in blue did not show significant abundance changes.

A pathway analysis summarizing the impact of *NAT1* KO on cellular energetics pathways is shown in Figure 10. Specifically, the biosynthesis pathways most impacted by *NAT1* KO were alanine, aspartate, and glutamate metabolism and the TCA/Krebs cycle. In Figure 11, we provide an illustrative outline of the glycolytic and TCA/Krebs cycle metabolites that highlights which intermediates were impacted by *NAT1* KO. To summarize, we observed that there were changes to fructose-6-phosphate and glyceraldehyde-3-phosphate, but the overall flux of glucose to pyruvate was unchanged. The ^13^C-enrichment into lactate and the flux of intermediates in the TCA/Krebs cycle were impacted by *NAT1* KO, as determined by the decreases in the ^13^C-enrichment of isocitrate, citrate, α-ketoglutarate, fumarate, and malate. In Figure 12, we provide a schematic figure outlining intermediates that had changes in their detected isotopologues for alanine, aspartate, and glutamate metabolism with *NAT1* KO. For alanine and aspartic acid, the metabolites affected by *NAT1* KO were aspartic acid, *L*-asparagine, and fumaric acid. For glutamic acid, the metabolites impacted by *NAT1* KO were glutamic acid and α-ketoglutarate. Figure 13 shows the metabolism pathway of arginine where the metabolites detected in the arginine metabolism pathways impacted by *NAT1* are known. Based on our results, the metabolites impacted in this pathway by the loss of *NAT1* were fumaric acid, aspartic acid, α-ketoglutaric acid, glutamic acid, and *N*-acetyl-glutamic acid.

Lastly, the data presented here are important, as they provide further evidence that *NAT1* knockout in breast cancer cells results in an altered metabolomic profile and provides additional evidence that NAT1 is related to proper mitochondrial functions in breast cancer cells. These data suggest that *NAT1* KO reductions in the growth of MDA-MB-231 breast cancer cells in vivo and in vitro may be due to disruptions in the cellular metabolism of glucose. Future work is warranted to understand upstream regulators of cellular energetics regulated by NAT1. These data also advance the idea that NAT1 should be further examined as a potential therapeutic target in breast cancer.

## Data Availability

Data provided in Supplementary Materials.

## Supplementary Materials

The following supporting information can be downloaded at: https://www.mdpi.com/article/10.3390/genes14040843/s1. Table S1. Positive Alignment Metabolites: All the metabolites derived from this dataset that had a positive alignment using a 2D LC-MS platform. Table S2. Negative Alignment Metabolites: All the metabolites derived from this dataset that had a negative alignment using a 2D LC-MS platform. Table S3. Statistical Results: statistical results of graphed metabolites (provided in the manuscript).

## Appendix A

### Detailed LC-MS Setting Information

All samples were analyzed by parallel two-dimensional liquid chromatography-mass spectrometry (2DLC-MS) composed of a Thermo Q Exactive HF Hybrid Quadrupole-Orbitrap Mass Spectrometer coupled with a Thermo DIONEX Ultimate 3000 HPLC system (Thermo Fisher Scientific, Waltham, MA, USA). The LC system was configured in parallel mode with a SeQuant ZIC^®^-cHILIC column (2.1 × 150 mm, 3 µm) as the hydrophilic interaction liquid chromatography (HILIC) column and a Waters Acquity UPLC HSS T3 column (2.1 × 150 mm, 1.8 µm) as the reversed phase chromatography (RPC) column. Each column was connected with a 2-μL sample loop. A sample was injected and separated in each of the two columns simultaneously. The eluant from the two columns was then mixed and delivered to the mass spectrometer. The temperature was set at 40 °C for the two columns.

For separation on the HILIC column, 10 mM ammonium acetate (pH 3.25) was used as mobile phase A, and 100% acetonitrile was used as mobile phase B. The flow rate was 0.3 mL/min. The gradient was: 0 min, 100% B; 0 to 5 min, 100% B to 35% B; 5 to 12.7 min, 35% B; 12.7 to 12.8 min, 35% B to 95% B; 12.8 to 14.3 min, 95% B. For separation on the RPC column, water with 0.1% formic acid was used as mobile phase A, and acetonitrile with 0.1% formic acid was used as mobile phase B. The flow rate was 0.4 mL/min. The gradient was as follows: 0 min, 0% B; 0 to 5 min, 0% B; 5 to 6.1 min, 0 to 15% B; 6.1 to 10 min, 15 to 60% B; 10 to 12 min, 60% B; 12 to 14 min, 60% to 100% B; 14 to 14.1 min, 100% to 5% B; 14.1 to 16 min, 5% B.

The parameters for mass spectrometry were as follows: the electrospray ionization probe fixed at level C, auxiliary gas 15 arbitrary units, auxiliary gas heater temperature 450 °C, capillary temperature 320 °C, sheath gas 55 arbitrary units, spray voltage 3.5 kV, sweep gas 3 arbitrary units, S-lens RF level 65.0, full scan range 140 to 1300 (*m*/*z*), resolution 30,000, maximum injection time 50 ms, and automatic gain control (AGC) 10^6^ ions.

Each biological sample was analyzed in random order in positive mode (+) and negative mode (−) to obtain the full MS data for metabolite relative quantification. The pooled samples were analyzed to acquire MS/MS spectra at different collision energies (20, 40, and 60 eV) for metabolite identification. The parameters for MS data acquisition were full scan from 60 to 900 (*m*/*z*), maximum injection time 50 ms, full MS resolution 30,000, and MS/MS resolution 15,000.
